# A cross-sectional study of olfactory and taste disorders among COVID-19 patients in China

**DOI:** 10.1186/s40779-021-00339-y

**Published:** 2021-09-13

**Authors:** Jian-Hui Li, Yi Sun, Mei-Rong Li, Hu Yuan, Chang-Liang Yang, Cheng-Cheng Huang, Feng-Jie Zhou, Rui-Yao Chen, Lei-Bo Zhang, Ning Yu, Qiong Liu, Jing-Jing He, Xue-Jun Zhou, Xiao-Bing Fu, Shi-Ming Yang

**Affiliations:** 1 College of Otolaryngology Head and Neck Surgery, Chinese PLA General Hospital, Beijing, 100853 China; 2 National Clinical Research Center for Otolaryngologic Diseases, No. 28 Fuxing Road, Beijing, 100853 China; 3 Department of Otolaryngology Head and Neck Surgery, Chinese PLA General Hospital Hainan Hospital, Sanya, 572013 China; 4 Hainan Clinical Research Center for Otolaryngologic Diseases, Sanya, 572013 China; 5 Department of Otolaryngology Head and Neck Surgery, General Hospital of Central Theater Command of PLA, Wuhan, 430070 China; 6 Research Center for Tissue Repair and Regeneration Affiliated to the Medical Innovation Research Division and 4th Medical Center, PLA General Hospital and PLA Medical College, Beijing, 100853 China; 7 PLA Key Laboratory of Tissue Repair and Regenerative Medicine and Beijing Key Research Laboratory of Skin Injury, Repair and Regeneration, No. 28 Fuxing Road, Beijing, 100853 China; 8 Research Unit of Trauma Care, Tissue Repair and Regeneration, Chinese Academy of Medical Sciences, Beijing, 100853 China

**Keywords:** COVID-19, SARS-CoV-2, Olfactory, Taste

## Abstract

To determine the prevalence and clinical features of olfactory and taste disorders among coronavirus disease 2019 (COVID-19) patients in China. A cross-sectional study was performed in Wuhan from April 3, 2020 to April 15, 2020. A total of 187 patients with confirmed severe acute respiratory syndrome coronavirus 2 (SARS-CoV-2) completed face-to-face interviews or telephone follow-ups. We found that the prevalence of olfactory and taste disorders was significantly lower in the Chinese cohort than in foreign COVID-19 cohorts. Females were more prone to olfactory and taste disorders. In some patients, olfactory and taste disorders precede other symptoms and can be used as early screening and warning signs.

**Supplementary Information:**

The online version contains supplementary material available at 10.1186/s40779-021-00339-y.

**Dear Editor,**

The spread of coronavirus disease 2019 (COVID-19) infection abroad has highlighted a novel and atypical set of symptoms: olfactory and taste disorders. It was not reported by most of the early studies from China and is still neglected in Chinese COVID-19 patients. Herein, we present the prevalence and clinical features of olfactory and taste disorders among COVID-19 patients in China.

A cross-sectional study was performed in Wuhan from April 3, 2020 to April 15, 2020. A total of 187 patients with confirmed severe acute respiratory syndrome coronavirus 2 (SARS-CoV-2) infection were admitted to the General Hospital of Central Theater Command of PLA in Wuhan, and face-to-face interviews or telephone follow-ups were completed. The demographic characteristics of olfactory and taste disorders are shown in Additional file 1: Table S1. The exclusion criteria were patients who were unable to independently complete the questionnaire, patients who had previous olfactory or taste disorders, and patients who were currently in the intensive care unit (ICU). A visual analog scale was used to score the patients’ olfactory status and taste. These data were also collected through online forms of discharged patients. SPSS version 22.0 was used to perform the statistical analysis.

The data analyses have led us to draw the following conclusions. Compared with the foreign cohort, the prevalence of smell and taste disorders in the Chinese cohort was significantly lower (Additional file 1: Table S1). The prevalence of olfactory and taste disorders in COVID-19 patients was higher in females than in males (Additional file 1: Table S1). In some patients, olfactory and taste disorders precede other symptoms and can be used as early screening and warning signs (Additional file 1: Table S2). The recovery of olfactory and taste function was independent of age; females have an easier recovery than males; olfactory or taste disorders were not easy to recover for those who are clinically classified as severe; when the olfactory or taste disorder itself was severe, it was not easy to recover; and olfactory or taste disorders that occurred in the early stage of the disease were more likely to recover than late-stage symptoms (Additional file 1: Table S3).

According to our research results, the incidence of olfactory and taste disorders in Chinese patients is markedly lower than that reported abroad, which is 64.4% [1]. The possible reason may be that the expression of the angiotensin-converting enzyme 2 (ACE2) receptor is different in the nasopharynx between East Asians and Europeans [2]. Increased expression of ACE2 in the nasopharynx may lead to an increased risk of olfactory and taste symptoms. The other possible reason is that the virus has mutated during the transmission process, or there are different strains. Different subtypes of viruses have different affinities and neurotoxicity to mucosal receptors in the olfactory and gustatory regions, resulting in differences in their biological behavior [3].

Olfactory disorders were significantly more prevalent among females than among males. The ACE2 gene is located on the X chromosome, so female individuals should have a higher ACE2 level [4], which might be the reason why they are more likely to be infected with SARS-CoV-2 than males. In addition, due to the effect of estrogen, women’s sense is more sensitive than men’s, and women's sense of smell fluctuations more obviously than men’s.

The pathophysiology of SARS-CoV-2’s influence on the olfactory and taste systems is unclear. Nevertheless, neuroinvasion through the olfactory sensory neurons (OSN) appears to be dominant because these cells are clearly the most susceptible to infection in the nasal cavity, and the olfactory bulb is the first central nervous system (CNS) tissue colonized upon intranasal inoculation [5]. Olfactory or taste disorders in the early stage of disease may trigger a series of early warning mechanisms to quickly mobilize the body's immune system to fight the virus’s attack, thereby preventing the virus from further attacking other important tissues and organs. Olfactory or taste disorders in the early stage of the disease may also mean that the host’s immune response is strong and effective, which is more conducive to the recovery of function.

Generally, there is no fever, cough or other symptoms in the early stage. These may lead to delays in diagnosis, treatment and further spread of the infections. We would consider olfactory or taste dysfunction as a possible early-warning symptom, especially in the absence of rhinitis. Compared with nucleic acid detection and chest CT, olfactory or taste disorder is a unique screening index because of its simplicity and low cost. Early identification of suspected patients, isolation monitoring, early diagnosis and treatment of COVID-19 patients are of great significance for China and other affected countries to conduct more accurate prevention and more efficient monitoring of COVID-19.

## Supplementary Information

**Additional file 1. Table S1**. Demographic characteristics of olfactory and taste disfunction in 187 COVID-19 patients.** Table S2**. Occurrence time and the corresponding proportion of personnel about olfactory and taste disorders.** Table S3**. Functional recovery vs. non-recovery in COVID-19 patients with olfactory or taste impairments.

## Data Availability

The datasets generated and analyzed during the current study are not publicly available due to individual privacy, but are available from the corresponding author on reasonable request.

## Abbreviations

ACE2: Angiotensin-converting enzyme 2
CNS: Central nervous system
COVID-19: Coronavirus disease 2019
ICU: Intensive care unit
OSN: Olfactory sensory neurons
SARS-CoV-2: Severe acute respiratory syndrome coronavirus 2
