# Supplementary material for: A cross-sectional study of olfactory and taste disorders among COVID-19 patients in China
Source: Mil Med Res. 2021 Sep 13;8:51. doi: 10.1186/s40779-021-00339-y (PMC8435759; doi:10.1186/s40779-021-00339-y)
Supplement: Supplementary file 1 — Additional file 1. Table S1. Demographic characteristics of olfactory and taste disfunction in 187 COVID-19 patients. Table S2. Occurrence time and the corresponding proportion of personnel about olfactory and taste disorders. Table S3. Functional recovery vs. non-recovery in COVID-19 patients with olfactory or taste impairments. [file 40779_2021_339_MOESM1_ESM.docx]

| Table S1 Demographic characteristics of olfactory and taste disfunction in 187 COVID-19 patients | | | | |
| --- | --- | --- | --- | --- |
| Item | Total | Olfactory  disfunction | Taste  disfunction | Olfactory and  taste disfunction |
| Number of patients[*n*(%)] | 187(100.00) | 23(12.30) | 42(22.46) | 22(11.76) |
| Age[years, median (IQR)] | 54(17-90) | 50(30-76) | 53(27-78) | 50(30-76) |
| Gender[*n*(%)] |  |  |  |  |
| Male | 95(50.80) | 7(3.74) | 17(9.10) | 7(3.74) |
| Female | 92(49.20) | 16(8.56) | 25(13.37) | 15(8.02) |

| Table S2 Occurrence time and the corresponding proportion of personnel about olfactory and taste disorders | | | | | |
| --- | --- | --- | --- | --- | --- |
| Time of  disorder | Olfactory disorders | |  | Taste disorders | |
|  | Number of people[*n*(%)] | Average number of days |  | Number of people[*n*(%)] | Average number of days |
| Before other symptom | 4(20.00) | -3.50 |  | 7(18.92) | -3.57 |
| At the same time | 2(10.00) | 0 |  | 4(10.81) | 0 |
| After other symptom | 14(70.00) | 7.50 |  | 26(70.27) | 9.65 |
| Total number | 20 | 4.55 |  | 37 | 6.38 |
| Twenty and thirty-seven patients with olfactory or taste disfunctions can clearly describe the fluctuation of symptoms. | | | | | |

| Table S3 Functional recovery *vs*. non-recovery in COVID-19 patients with olfactory or taste impairments | | |
| --- | --- | --- |
| Item | Recovery | Non-recovery |
| Number of patients | 33 | 9 |
| Age[years, median (IQR)] | 54(30-76) | 54(41-61) |
| Gender[*n*(%)] |  |  |
| Male | 13(39.39) | 4(44.44) |
| Female | 20(60.61) | 5(55.56) |
| Clinical severe[*n*(%)] | 8(24.24) | 4(44.44) |
| Moderate or severe impairment of olfactory or taste[*n*(%)] | 5(15.15) | 3(33.33) |
| Time of disorder after other symptom (d) | 2.73 | 27.40 |
